# High‐metacarpal deep digital flexor tenotomy and Steward clog shoeing for managing chronic refractory laminitis: A retrospective clinical study

**DOI:** 10.1111/vsu.70068

**Published:** 2025-12-14

**Authors:** Kristyna Hargitaiova, Grigorios Maleas

**Affiliations:** ^1^ College of Veterinary Medicine Cornell University Ithaca New York USA; ^2^ Equuria Orthopedics GbR Emstek Germany

## Abstract

**Objective:**

To describe outcomes following high‐metacarpal deep digital flexor tenotomy (DDFT) combined with Steward clog application in horses and ponies with refractory chronic laminitis.

**Study design:**

Retrospective observational study.

**Animals:**

Client‐owned horses and ponies (7 horses, 8 ponies) with severe refractory laminitis.

**Methods:**

Medical records (2018–2019) were reviewed. All underwent standing high‐metacarpal DDFT tenotomy proximal to the accessory ligament (AL‐DDFT) junction, followed by customized Steward clogs application. Preoperative assessment included radiography and venography. Postoperative management involved nonsteroidal anti‐inflammatory drugs (NSAIDs), controlled exercise, and serial monitoring of comfort, alignment, and survival up to 24 months.

**Results:**

Median follow‐up was 24 months (range: 6–24 months). Six‐month survival was 100% (7/7) in horses and 88% (7/8) in ponies. At 12 months, 43% (3/7) of horses and 88% (7/8) of ponies remained alive and improved to Obel grades 0–2. At 24 months, 43% (3/7) of horses and 50% (4/8) of ponies survived, one returning to light work. Obel grades at 24 months ranged from 0 to 2 (Obel 0: *n* = 3, Obel 1 = 3, Obel 2 = 1). A single distal interphalangeal joint (DIPJ) subluxation (4%, 1/26 limbs) resolved with corrective farriery. Target palmar angles (3°–10°) were achieved in all cases. The majority of non‐survivors had endocrine‐associated laminitis.

**Conclusion:**

High‐metacarpal DDFT tenotomy, with preservation of the AL‐DDFT, combined with Steward clog application provided effective P3 realignment and lameness improvement with low DIPJ subluxation incidence.

**Clinical significance:**

This technique maintained DIPJ stability in 25/26 joints and achieved outcomes comparable to previously described tenotomy methods.

## 1 INTRODUCTION

Laminitis remains one of the most painful and debilitating equine conditions, especially in refractory cases, a frequent cause of euthanasia.^1^, ^2^ It results from failure of the dermal‐epidermal attachment between the distal phalanx (P3) and hoof capsule, leading to mechanical instability, vascular compromise, and severe pain.^3^, ^4^ Severe Obel grade lameness, distal displacement of the P3 (“sinking”), and concurrent systemic disease are strong predictors of poor outcome and in‐hospital mortality.^1^, ^2^, ^5^ Horses with endocrinopathic laminitis, particularly due to equine metabolic syndrome (EMS) and/or pituitary pars intermedia dysfunction (PPID), often show variable therapeutic responses and are prone to recurrence.^6^

Conservative treatment involves stall confinement, solar and frog support, corrective trimming and therapeutic shoeing, analgesia, digital cryotherapy, dietary management, and close clinical and radiographic monitoring.^7^ Conservative approaches are considered to have failed when pain, radiographic displacement, or collapse of hoof capsule mechanics progresses despite appropriate management.^1^, ^8^ In such cases, deep digital flexor tendon (DDFT) tenotomy may be performed as a salvage procedure to reduce tension on the P3, facilitate mechanical realignment of the P3, and improve comfort.^9^, ^10^

Two principal DDFT tenotomy sites have been described: a mid‐metacarpal approach and a distal (pastern) approach.^9^, ^10^, ^11^ Both reduce DDFT tension, but excessive release can lead to distal interphalangeal joint (DIPJ) hyperextension or instability, particularly following distal transection. Cadaveric studies confirmed altered distal joint angles following either technique, with greater DIPJ extension observed at the pastern‐level transection.^12^

The accessory ligament of the DDFT (AL‐DDFT) provides passive restraint against carpal and fetlock overextension and contributes to DIPJ stability.^13^ Preserving AL‐DDFT may therefore help maintain distal limb alignment following DDFTtransection. The modified high‐metacarpal DDFT tenotomy evaluated here was designed to achieve adequate reduction of DDFT tension while retaining the AL‐DDFT attachment, aiming to preserve postoperative DIPJ stability.

Even with preservation of the AL‐DDFT, postoperative DIPJ instability and uneven weight distribution may occur without adequate mechanical support. These can be mitigated through postoperative farriery, including heel support and caudal extension to restore the P3 alignment and reduce the moment arm acting on the DDFT.^14^ Supporting the heel with padding reduces the moment arm of the DDFT acting on the P3; in other words, it shortens the distance between the tendon's line of pull and the DIPJ, thereby decreasing the leverage that promotes DIPJ hyperextension. Steward clogs are wooden, weightbearing orthotic shoes that incorporate a flat wooden base and soft impression material to redistribute load across the sole and provide mechanical stabilization of the distal limb.^15^ They have been widely adopted by veterinarians and farriers in chronic laminitis cases and allow easy shoe base modification to adjust palmar angle and optimize P3 alignment.^15^

This study retrospectively evaluated outcomes of a modified high‐metacarpal DDFT tenotomy technique that preserves the AL‐DDFT, combined with Steward clog application in horses and ponies with refractory chronic laminitis. The objectives were to: (1) document short‐ and long‐term lameness outcomes and survival, and (2) evaluate whether preserving the AL‐DDFT reduced the risk of DIPJ subluxation.

## 2 MATERIALS AND METHODS

### 2.1 Case selection and preoperative evaluation

This retrospective observational study reviewed medical records of client‐owned horses and ponies treated between January 2018 and January 2019. Extracted data included signalment, laminitis etiology, duration, prior treatments, pre‐ and postoperative radiographic parameters (palmar angle, rotation, sole depth), venographic findings, complications, and lameness grades (Obel and American Association of Equine Practitioners [AAEP]) at 3, 6, 12, and 24 months. The primary outcome was survival to 24 months post‐tenotomy; secondary outcomes were lameness improvement, radiographic realignment, and complication incidence.

All horses underwent standardized clinical and imaging evaluation before surgery. Lameness severity was graded using the Obel system. Weightbearing lateromedial and dorsopalmar radiographs and digital venograms were obtained following established techniques.^16^, ^17^ Venograms were assessed for reduced perfusion of the terminal arch, solar plexus, or dorsal lamellar vessels. In unilateral cases, the contralateral forelimb was imaged to confirm disease localization.

Inclusion criteria comprised severe unilateral or bilateral chronic laminitis unresponsive to medical and farriery management. Horses with >11.8 mm dorsal separation^18^ or marked vascular compromise of the solar plexus or terminal arch were excluded and humanely euthanized for welfare reasons. Only horses meeting these criteria underwent surgery and were included in analyses.

As a retrospective review of clinical cases involving client‐owned animals, this study did not require institutional animal care and use approval.

### 2.2 Surgical technique

All procedures were performed in standing horses under sedation and local anesthesia. The AL‐DDFT junction was identified preoperatively using ultrasonography, allowing precise localization. Intraoperatively, the tenotomy was performed proximal to this junction, ensuring that the AL‐DDFT remained intact. Detomidine hydrochloride (0.01 mg/kg IV, Domidine, Dechra Veterinary Products Deutschland GmbH, Düsseldorf, Germany) and butorphanol tartrate (0.01 mg/kg IV, Torbugesic, Zoetis Deutschland GmbH, Berlin, Germany) were administered, followed by aseptic preparation of the proximal metacarpal region. A high subcutaneous ring block (Zone 1A) was performed with 5 mL of 2% mepivacaine hydrochloride (Intra‐Epicaine, Dechra Veterinary Products Deutschland GmbH, Düsseldorf, Germany), and 3 mL was infiltrated at the proposed transection site.

**FIGURE 1:**
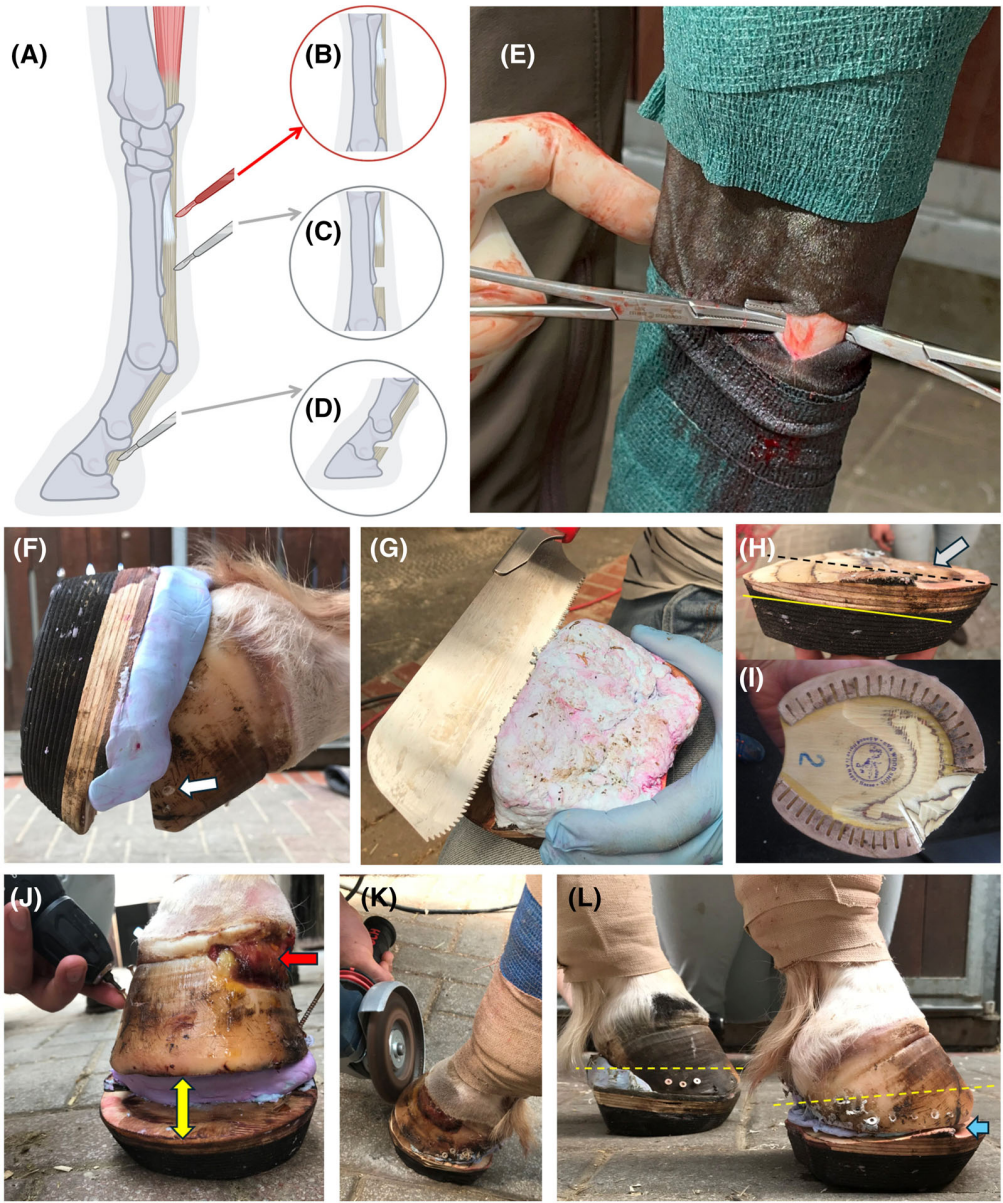
High‐metacarpal deep digital flexor tenotomy (DDFT) tenotomy and postoperative Steward clog application. (A–D) Schematic comparison of tenotomy sites. (B) High‐metacarpal approach (this study) performed proximal to AL‐DDFT versus (C) mid‐metacarpal and (D) distal/pastern‐level techniques. (E) Isolation of the DDFT using curved hemostats before transection. (F) Application of hoof putty (LUVEX Hufrehe‐Polster) with predrilled anchoring holes (white arrow). (G) Trimming of hardened putty height to restore hoof‐ground alignment. (H–I) Customization of Steward clogs to offload affected regions—lowering the palmar region (black dashed vs. yellow line) and leaving the solar area unweighted (gray arrow). (J) Clog attachment with screws; unloaded solar prolapse (yellow arrow), debrided coronary band from abscessation indicated (red arrow). (K) Clog shaping with a grinder. (L) Final stage before casting; yellow dashed lines denote cast border.

A 2–3 cm longitudinal skin incision was made on the lateral proximal metacarpus at the junction of Zones 1B–2A. Subcutaneous tissues and deep fascia were incised using Metzenbaum scissors. The superficial digital flexor tendon (SDFT), DDFT, and AL‐DDFT were identified and the mesotenon was bluntly separated. Curved Kelly hemostats were placed palmar and dorsal to the DDFT to protect the neurovascular structures before exteriorizing the DDFT (Figure 1E). The DDFT was sharply transected with a #10 scalpel blade proximal to the AL‐DDFT junction. Complete transection was confirmed visually and by palpation. The integrity of the AL‐DDFT was confirmed visually and by digital palpation of the preserved check ligament fibers before closure. The site was lavaged with hypochlorous acid solution (Vetericyn Plus VF), closed with 2–0 or 3–0 nylon in simple interrupted pattern, and bandaged. Systemic antimicrobials were administered only when sole penetration or contamination was present.

In cases with coronary band abscessation, debridement with or without hoof wall resection was performed as described by Eustace et al.^18^ In horses without active drainage, dorsal hoof‐wall groove just distal to the coronary band was created to promote perfusion and stimulate new wall growth, as described by Ritmeester et al.^19^

### 2.3 Postoperative farriery

Immediately after surgery, excess dorsal hoof wall was trimmed, and Steward clogs were applied as previously described (Steward).^15^ After hoof preparation, the quarters were drilled from distal to proximal, and a soft impression material (LUWEX Hufrehe‐Polster Premium) was applied to the palmar sole for support (Figure 1F,G). Clogs were customized according to radiographic and venographic findings to relieve pressure over affected regions (Figure 1H,I). The clogs were secured using 3.5‐mm full‐thread, zinc‐plated Torx (T15) wood screws (chipboard screws), 40 mm in length, (REYHER, Hamburg, Germany). The screws were inserted through the predrilled holes while weightbearing and secured into the prefabricated wooden base, which was then shaped to fit the hoof (Figure 1J,K). In cases with sole penetration, the toe region was left non‐weightbearing and packed with iodine‐impregnated gauze before bandaging. A thermoplastic cast (Vetcast Plus) was molded around the hoof and clog to secure fixation and protect screw heads.

**FIGURE 2:**
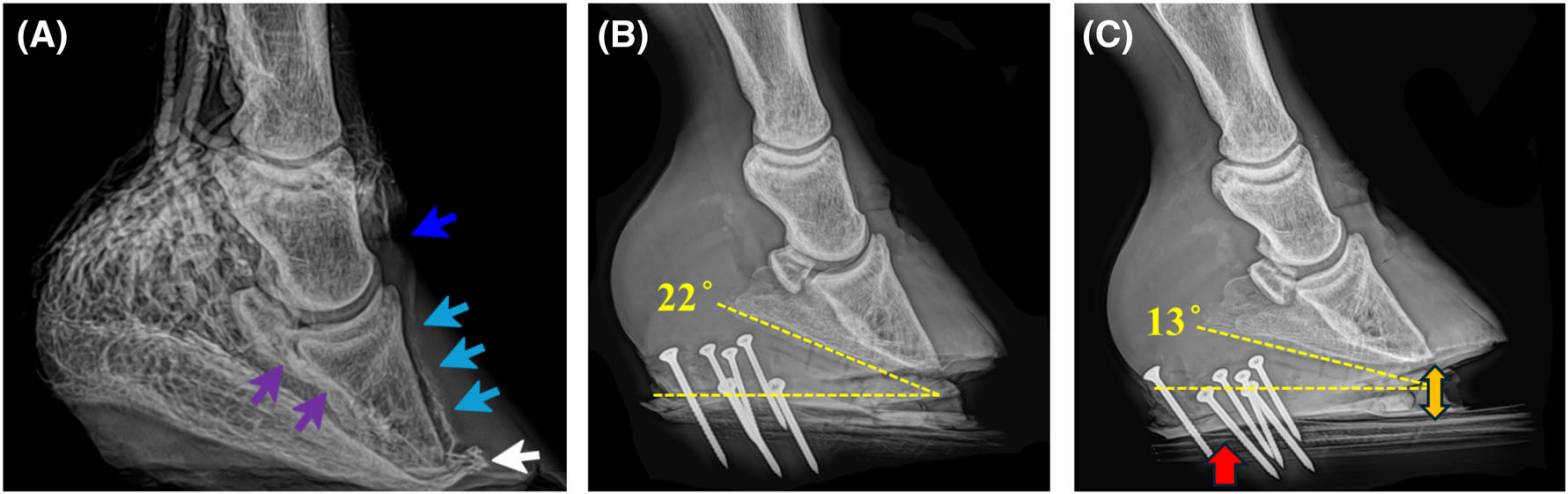
Radiographic adjustment of palmar angle in a horse with solar penetration. (A) Preoperative venogram showing marked loss of coronary plexus perfusion (dark blue arrow), dorsal lamellar plexus (light blue arrows), terminal papillae compression (white arrow) and reduced terminal arch (purple arrows). This sole was penetrated. (B) Immediate postoperative radiograph with a 22° palmar angle and persistent pressure on the tip of P3. (C) The palmar surface of the clog was rasped (red arrow) to reduce the angle to 13°, creating clearance beneath the P3 apex (yellow arrow) to relieve solar compression and improve comfort.

A standing lateromedial radiograph was obtained postoperatively to confirm clog position and target palmar angle (3°–6°). In low‐heeled horses, the palmar surface of the clog was trimmed incrementally until alignment was achieved radiographically (Figure 2).

### 2.4 Postoperative management and follow‐up

Horses were stall‐confined for 1–2 days postoperatively, then hand‐walked three times dailyfor 3–5 min per session. Walking duration was incrementally increased by approximately 5 min per day each week, based on individual comfort and clinical assessment. Nonsteroidal anti‐inflammatory drugs (phenylbutazone 2.2–4.4 mg/kg orally every 12–24 h or flunixin meglumine 1.1 mg/kg orally/IV every 12–24 h) were administered until comfortable ambulationIn cases with sole penetration, iodine‐impregnated packing was changed every 2 days. Skin sutures were removed after 12–14 days.

Steward clogs were replaced every 4–6 weeks, with radiographs obtained at each change to monitor P3 alignment and sole depth until sufficient sole thickness developed. When comfort and radiographic improvement were achieved, horses were transitioned to therapeutic shoeing under farrier supervision. Control radiographs were obtained every 6 months thereafter.

Gait was evaluated by the surgeon (GM) at 3 and 6 months and by referring veterinarians at 12 and 24 months. Lameness was graded using the Obel system (I–IV) throughout and the AAEP scale (0–5) from 6 months onward.

### 2.5 Statistical analysis

Descriptive statistics were generated (proportions, medians, minimum‐maximum values). Kaplan–Meier survival analysis was performed to estimate survival probability at 6, 12, and 24 months. Given the small sample size and heterogeneity, no inferential statistics were applied.

## 3 RESULTS

### 3.1 Case population

**FIGURE 3:**
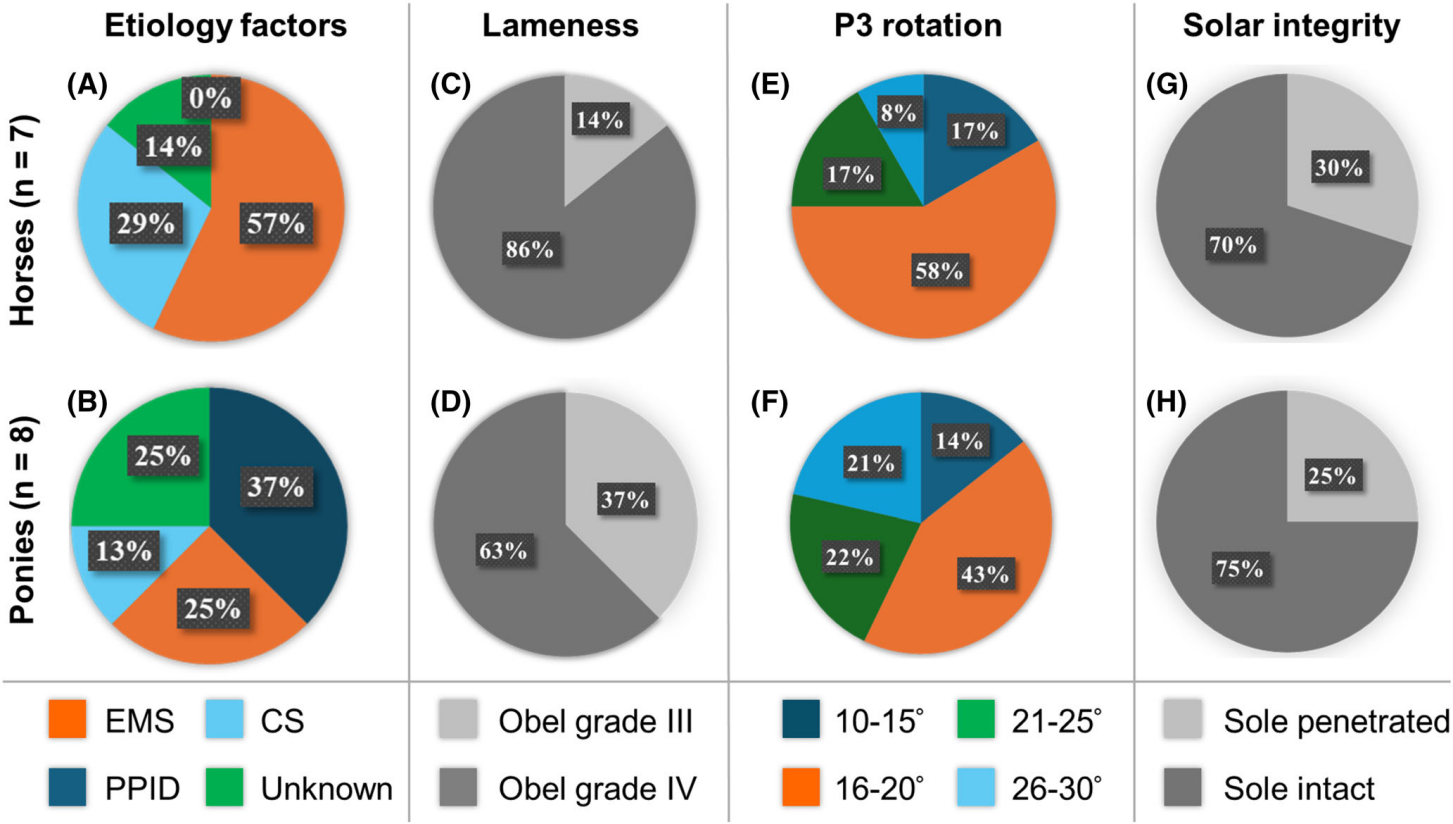
Preoperative case characteristics in horses (top) and ponies (bottom). Etiology, lameness grade, degree of P3 rotation, and solar integrity are shown. (A) Endocrine‐associated laminitis was prevalent in horses (equine metabolic syndrome [EMS] 4/7, 57%) with 2/9 (29%) of horses receiving corticosteroids. Ponies (B) showed a more even distribution (EMS 2/8, 25%, pituitary pars intermedia dysfunction [PPID] 3/8, 37%) 1/8 (13%) received corticosteroids and etiology was unknown in 2/8 (25%). (C, D) Most horses (86%) and ponies (63%) presented with Obel grade IV lameness. (E, F) Moderate P3 rotation (15°–20°) was most common (horses 58%, ponies 43%), while >25° rotation occurred in 8% and 14%, respectively. (G, H) Solar penetration was identified in 30% of horses and 25% of ponies.

A total of 15 horses and ponies met the inclusion criteria between January 2018 and January 2019 (Table 1; Figure 3). The cohort comprised 11 geldings and four mares, aged 7–29 years (median 13 years) and weighing 112–725 kg (median 401 kg). For descriptive analysis, animals were grouped as horses (*n* = 7) and ponies (*n* = 8).

**TABLE 1:** Signalment and clinical presentation in 15 horses and ponies undergoing DDFT tenotomy for chronic laminitis.

Horse	Breed	Age (years)	Sex	Weight (kg)	Limb	Obel Grade	Etiology	Duration	P3 rotation (°)	Sole penetration
1	Warmblood	16	F	563	LF, RF	IV	Corticosteroids (IA)	11 weeks	LF = 17 RF = 20	Yes
2	Haflinger	8	F	598	LF, RF	III	EMS	1 year	LF = 16 RF = 17	No
3	Warmblood	13	F	433	LF	IV	Corticosteroids (S)	4 weeks	LF = 12	Yes
4	Warmblood	12	F	648	LF, RF	IV	EMS	6 weeks	LF = 20 RF = 21	No
5	Warmblood	16	F	592	LF, RF	IV	Unknown	13 weeks	LF = 18 RF = 22	No
6	Quarter Horse	12	G	469	LF, RF	IV	EMS	9 weeks	LF = 15 RF = 17	No
7	Friesian	7	F	725	RF	IV	EMS	17 weeks	RF = 30	Yes

Pony	Breed	Age (years)	Sex	Weight (kg)	Limb	Obel Grade	Etiology	Duration	P3 rotation (°)	Sole penetration
1	German riding pony	13	G	368	LF, RF	IV	PPID	8 weeks	LF = 19 RF = 13	Yes
2	Icelandic horse	12	G	340	RF	IV	Corticosteroids (S)	4 weeks	RF = 19	Yes
3	German riding pony	21	G	371	LF, RF	IV	PPID	18 weeks	LF = 21 RF = 15	No
4	Shetland	11	F	112	LF	III	EMS	2 years	LF = 23	No
5	Welsh pony	29	G	280	LF, RF	IV	PPID	8 years	LF = 27 RF = 30	No
6	Shetland pony	16	F	135	LF, RF	IV	EMS	2 years	LF = 19 RF = 17	No
7	German riding pony	18	G	401	LF, RF	III	Unknown	9 weeks	LF = 16 RF = 18	No
8	German riding pony	19	G	270	LF, RF	III	Unknown	31 weeks	LF = 26 RF = 24	No

*Note*: Breed, age, sex, weight, affected limb(s), Obel grade, underlying etiology, disease duration, degree of P3 rotation, and presence of solar penetration are shown for each case.

Abbreviations: DDFT, deep digital flexor tenotomy; EMS, equine metabolic syndrome; F, female; G, gelding; IA, intra‐articular corticosteroid; LF, left forelimb; PPID, pituitary pars intermedia dysfunction; RF, right forelimb; S, systemic corticosteroid.

Bilateral forelimb laminitis was present in 71% (5/7) horses and 75% (6/8) ponies; unilateral disease occurred in 29% (2/7) horses and 25% (2/8) ponies. Corticosteroid administration (dexamethasone or triamcinolone) within 4 weeks of onset was documented in 29% (2/7) horses and 13% (1/8) ponies. An underlying endocrinopathy (EMS or pituitary pars intermedia dysfunction [PPID]) was identified in 57% (4/7) horses and 62% (5/8; 25%–2/8 EMS, 37%–3/8 PPID) ponies, while no clear cause was found in 13% (1/7) horses and 25% (2/8) ponies.

Duration of laminitis before surgery ranged from 4 weeks to 8 years (median 9 weeks). All animals had received phenylbutazone or flunixin meglumine for at least 3 weeks, and multiple corrective shoeing methods (reverse, egg‐bar, wedge, clog, or pad configurations) had been attempted without sustained improvement.

### 3.2 Preoperative findings and concurrent procedures

Radiography confirmed distal phalanx rotation >12° in all cases, ranging from 12° to 30° (mean 19.7°; Table 1, Figure 3). Sole penetration ‐ defined as protrusion of the solar corium (five hooves) or visible P3 apex (one hoof) ‐ occurred in 23% (6/26) affected feet, including 25% (2/8) ponies and 30% (3/7) horses. In cases with sole penetration and coronary‐band drainage (5/26), coronary‐band debridement was performed, with proximal dorsal hoof‐wall resection in one horse (Table 2). Horn regrowth was evident within 2–4 weeks in all six cases. In the remaining case, a dorsal hoof‐wall groove was created to promote revascularization.

**TABLE 2:** Postoperative course, complications, and 24‐month outcomes in 15 horses and ponies following DDFT tenotomy.

Horse	Dorsal wall resection	Postoperative complications	3 m postoperative (Obel grade)	6 m postoperative (Obel grade/AAEP)	12 m postoperative (Obel grade/AAEP)	24 m postoperative (Obel grade/AAEP)
1	Yes	None	III	II/4	IV (euthanized)	
2	No	None	II	II/4	I/4	II/4
3	Yes	None	0	0/3	0/2	0/0
4	No	None	II	I/4	IV (euthanized)	
5	No	None	I	I/4	IV (euthanized)	
6	No	None	II	II/4	I/4	I/4
7	Yes	None	0	0/3	IV (euthanized)	

Pony	Dorsal wall resection	Postoperative complications	3 m postoperative (Obel grade)	6 m postoperative (Obel grade/AAEP)	12 m postoperative (Obel grade/AAEP)	24 m postoperative (Obel grade/AAEP)
1	Yes	Wound dehiscence (LF) DIPJ subluxation (LF)	II	II/4	0/0	0/0
2	Yes	None	0	0/3	0/2	0/2
3	Yes	Seroma (RF)	0	0/2	0/2	IV (euthanized)
4	No	None	I	II/4	I/4	IV (euthanized)
5	No	Seroma (LF, RF)	II	II/4	II/4	IV (euthanized)
6	No	None	III	IV (euthanized)		
7	No	None	I	I/4	I/4	I/4
8	No	None	II	I/4	I/4	I/4

*Note*: Dorsal hoof wall resection, complications, and serial Obel and AAEP lameness grades (3–24 months) are shown for each case. Individuals euthanized for persistent or recurrent lameness are shaded in gray.

Abbreviations: AAEP, American Association of Equine Practitioners lameness scale (0–5); DIPJ, distal interphalangeal joint; LF, left forelimb; RF, right forelimb.

### 3.3 Surgical and postoperative complications

Surgical and immediate postoperative complications were minimal (Table 2, Figure 5A). Seroma formation occurred in 2/26 (8%) limbs and resolved after drain placement. One wound dehiscence (4%, 1/26) developedtwo days after suture removal and healed by secondary intention. All three wound complications occurred in horses with PPID. No surgical site infections required systemic antimicrobial therapy.

DIPJ subluxation developed in 4% (1/26) of hooves at 4 weeks postoperatively. Pony 1 presented with a dorsal capsular rotation of 19°. At the time subluxation was detected, the digital axis deviation measured 156°, which improved to 176° following Steward clog adjustment to extend the heel support. The subluxation resolved within 2 weeks, and no additional DIPJ hyperextension or subluxation cases were observed during follow‐up.

### 3.4 Short‐term outcomes (6 months)

**FIGURE 4:**
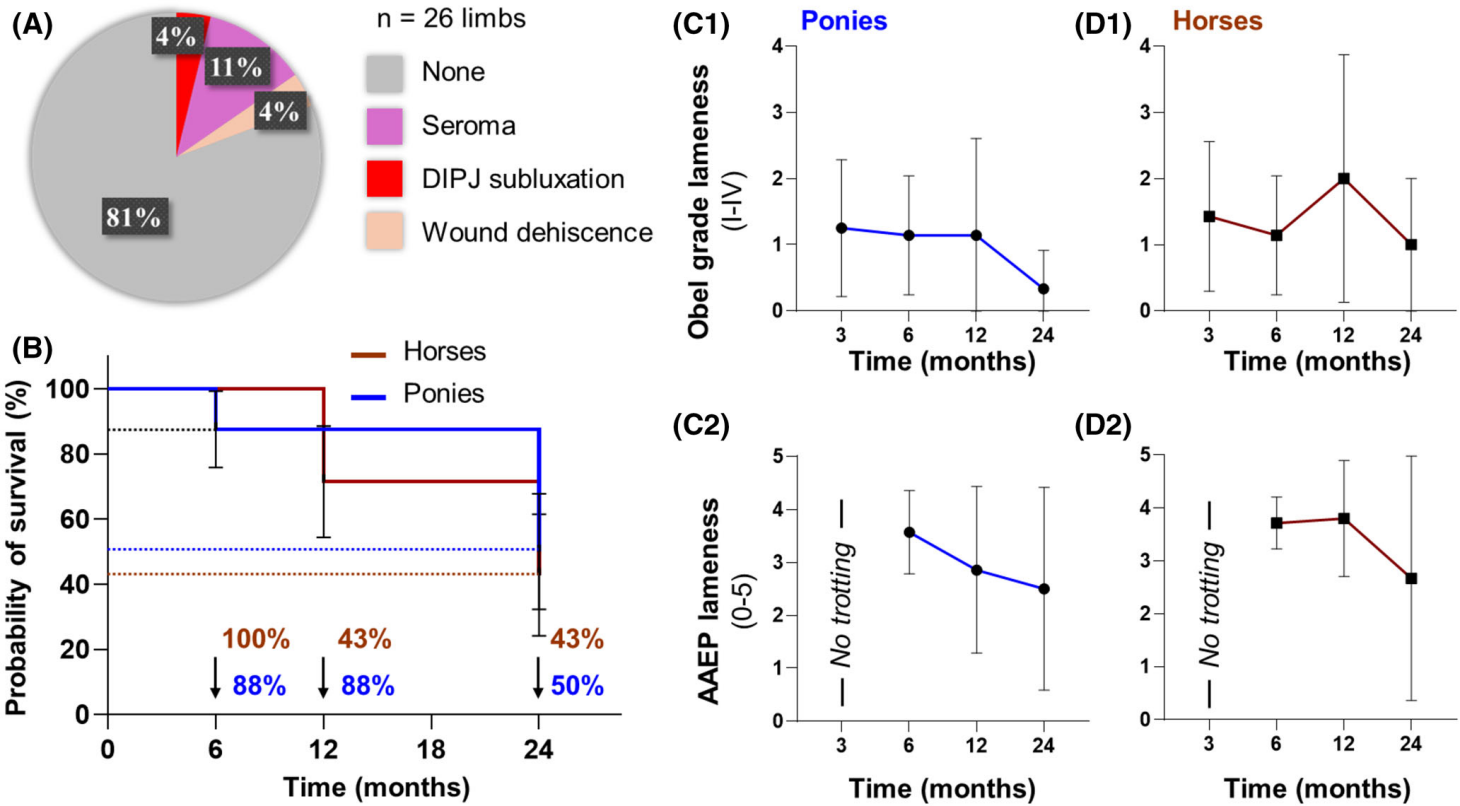
Postoperative complications and clinical outcomes. (A) Most limbs (21/26, 81%) had no complications; minor events included seroma formation (3/16, 12%), wound dehiscence (1/26, 4%), and transient distal interphalangeal joint (DIPJ) subluxation (1/26, 4%). (B) Kaplan–Meier analysis showing 24‐month survival: 100% short‐term survival in horses and 88% in ponies, declining to 43% and 50%, respectively, due to relapse or late euthanasia severe or recurrent cases. (C) Both groups showed improved soundness over time, with surviving horses and ponies achieving mean AAEP grades 2–3 and Obel grades 0–1 by 12–24 months. Horses and ponies were not trotted at 3 months; therefore, AAEP scores are not reported at that time. Data are shown as mean ± SD; statistical comparisons were not performed due to small sample size.

At 6 months, 100% (7/7) horses and 88% (7/8) ponies remained alive. Clinical improvement ‐ defined as a reduction of at least two lameness grades (AAEP or Obel) ‐ was achieved in all survivors except one pony, which was euthanized due to persistent lameness and client's preference (Figure 4C,D; Table 2). Among horses, two were sound at walk and 3/5 AAEP lame at trot, two showed Obel grade I, and three had Obel grade II. Of the ponies, two were sound at walk and AAEP 2–3/5 at trot, two exhibited Obel I and three Obel II lameness.

### 3.5 Long‐term outcomes (12–24 months)

At 12 months postoperatively, 43% (3/7) horses and 88% (7/8) ponies were alive (Figure 4B). Four horses were euthanized between 6 and 12 months due to progressive or recurrent laminitis unresponsive to continued management. Lameness grades among 12‐month survivors ranged from sound‐at‐walk to Obel grade II or AAEP grade 2/5 in unilateral cases.

At 24 months, 43% (3/7) horses and 50% (4/8) ponies were alive. (Figure 4B). All survivors maintained good body condition; two horses and three ponies were sound at walk or mildly lame (Obel I–II), and one pony had returned to light riding work (Figure 4C,D, Table 2).

### 3.6 Influence of underlying disease and laterality

Among horses, two survivors (67%, 2/3) and two non‐survivors (50%, 2/4) had endocrinopathic laminitis (underlying EMS). Among ponies, endocrinopathy (underlying EMS or PPID) was present in all non‐survivors (100%, 4/4) and one (25%, 1/4) survivor. At 24 months, survival was similar between bilateral and unilateral cases: 40% (2/5) horses and 50% (3/6) ponies with bilateral laminitis survived, compared with 50% (1/2) horses and 50% (1/2) ponies with unilateral disease. (Tables 1 and 2). Small sample size precluded statistical analysis.

### 3.7 Radiographic outcomes

Target realignment of P3 (palmar angle 3°–10°) was achieved in all 26 treated hooves within 6 weeks postoperatively and maintained throughout the 24‐months follow‐up in survivors. Progressive sole depth improvement was evident radiographically, with adequate thickness (15–20 mm) restored within 3–6 months.

**FIGURE 5:**
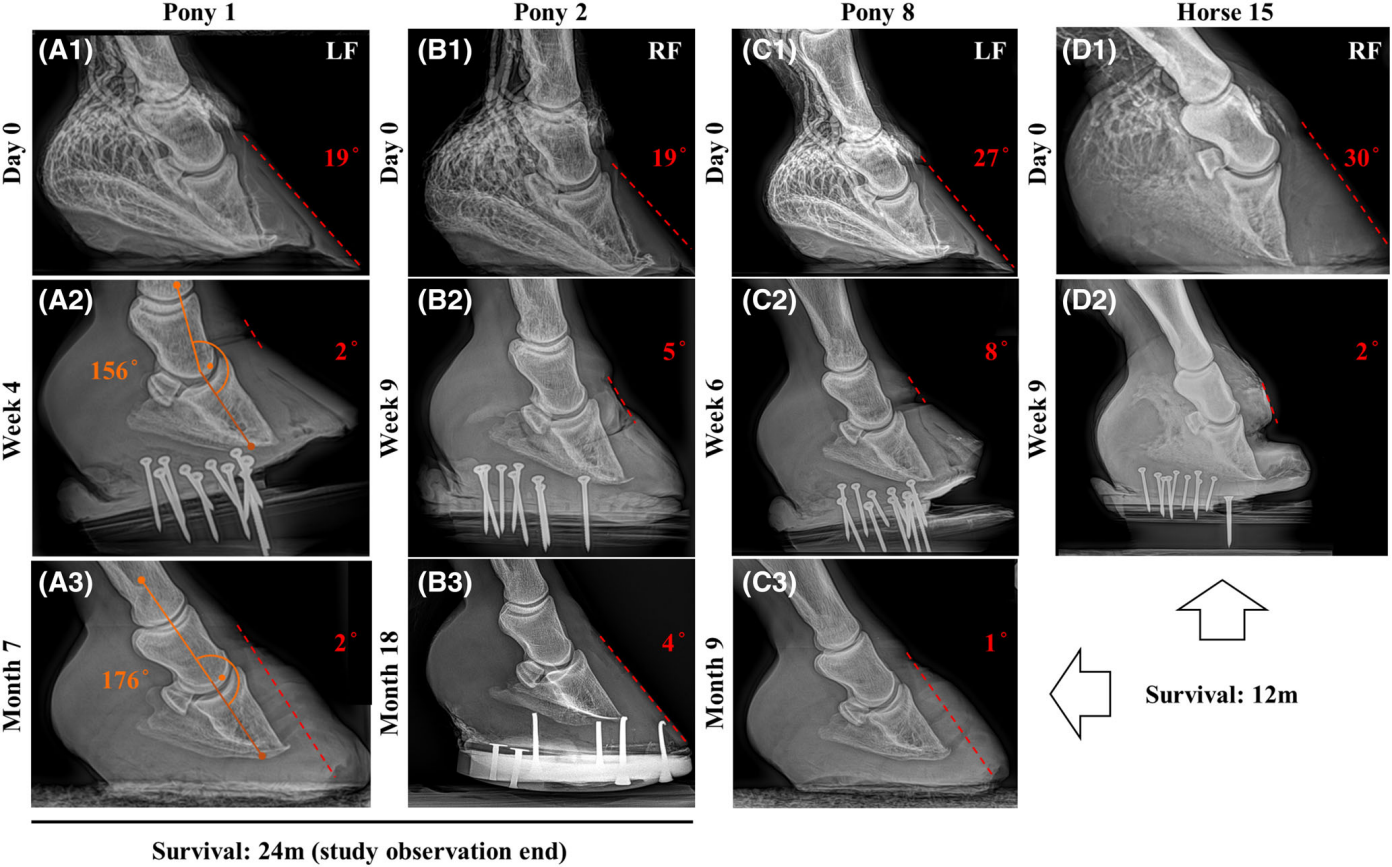
Postoperative radiographic progression. (A–D) Sequential radiographs of four representative horses with varying degrees of P3 rotation and vascular compromise. Preoperative venograms (A1–D1) show differing levels of perfusion deficit, most pronounced in the coronary plexus and dorsal lamina. In Horse 15 (D1), venographic contrast was poor due to suboptimal technique, but severe ischemia associated with the advanced 30° rotation could not be excluded. Postoperative radiographs (A2–D2) demonstrate improving P3 realignment and stabilization. Ponies 1 and 2 (A, B) had <20° rotation and survived to 24 months; Pony 1 developed a transient distal interphalangeal joint (DIPJ) subluxation at 4 weeks, at which time the digital axis deviation measured 156°; following heel extension adjustment, the axis improved to 176° by 7 months and remained stable thereafter. Pony 8 and Horse 15 (C, D) presented with greater rotation (27° and 30°, respectively); Pony 8 realigned but remained lame and was euthanized within 12 months, whereas Horse 15 initially improved but relapsed at 12 months. Final follow‐up (A3–C3) shows maintained palmar angle and progressive hoof capsule realignment in short‐ and long‐term survivors.

Preoperative venograms showed variable perfusion deficits, most often involving the coronary plexus and dorsal laminar vasculature (Figure 5). Compression of the solar plexus by the displaced P3 was noted in several cases. Horses with milder P3 rotation (<20°) achieved more consistent realignment and long‐term survival, whereas those with advanced rotation (>25°) had poorer outcomes.

Pony 1 developed transient DIPJ subluxation 4 weeks postoperatively, which resolved after hoof balance adjustment and was radiographically corrected by 7 months. Pony 8 and Horse 15, presenting with severe 27° and 30° rotation, respectively, initially improved radiographically but relapsed within 12 months and were euthanized due to recurrent lameness.

## 4 DISCUSSION

High‐metacarpal DDFT tenotomy with preservation of the AL‐DDFT, combined with Steward clog support yielded 12‐month survival of 43% (3/7) in horses and 88% (7/8) in ponies and a 24‐month survival of 43% (3/7) in horses and 50% (4/8) in ponies, with few complications in severely affected, refractory cases. These outcomes are broadly consistent with historical reports of DDFT tenotomy as a salvage procedure. In a 35‐horse retrospective cohort, Eastman et al.^10^ reported 77% survival at 6 months and 59% at ~2 years after mid‐metacarpal DDFT tenotomy, highlighting similar long‐term expectations for closely managed cases. A larger, more recent study by Morrison^21^ reviewed 245 DDFT tenotomy cases with standardized realignment shoeing and reported a 51% long‐term success (>12 months), with survival exceeding 80% in cases lacking sinking or severe bone disease.

The rare incidence of DIPJ subluxation (4%; 1/26 limbs) might reflect both the proximal transection level and the immediate, mechanically targeted orthotic support. DIPJ subluxation has often been discussed anecdotally following DDFT tenotomy but is rarely documented in retrospective series. Morrison^21^ described mild radiographic subluxation as a normal postoperative finding that typically resolved with subsequent shoeings, while most previous studies did not specifically report its incidence. In contrast, the present series recorded subluxation as a defined clinical and radiographic outcome, identifying only a single transient case that resolved after hoof balance adjustment.

Recent experimental and clinical refinements in tenotomy technique have focused on achieving controlled tendon unloading while minimizing iatrogenic instability. Zetterström et al.^21^ evaluated partial DDFT transection (double hemitenotomy) to preserve residual tensile integrity, and De Gasperi et al.^22^ introduced an ultrasound‐guided percutaneous thread‐transection method to reduce surgical morbidity. Although neither directly assessed DIPJ alignment, both reflect a broader shift toward partial or minimally invasive DDFT release aimed at controlled mechanical decompression without excessive loss of support.

In the present study, preservation of the continuity of the accessory ligament with its strong proximal attachment to the distal carpal row via the palmar carpal ligament^13^ together with immediate orthotic realignment, might have contributed to the exceptionally low rate of DIPJ displacement, although further biomechanical studies are warranted.

The use of Steward clogs offered several practical and therapeutic advantages. Their non‐invasive, weightbearing application minimizes limb elevation time and reduces strain on the contralateral foot ‐ an important welfare consideration in bilateral cases.^15^ Unlike glue‐on shoes, which require prolonged elevation and often necessitate nerve blocks in the supporting limb,^20^ clogs can be rapidly secured without anesthesia. The combination of a wooden base and soft impression material provides effective shock absorption and uniform load distribution across the sole,^23^ promoting comfort and early mobilization. Moreover, simple rasping adjustments to the palmar or dorsal surface allow precise modification of the digit axis without clog removal, facilitating dynamic alignment corrections. While highly adaptable and well tolerated in this cohort, the authors acknowledge that Steward clogs may be less durable in heavier breeds and require practitioner familiarity for optimal customization.

Endocrinopathic laminitis was common in this cohort and, consistent with prior reports,^2^, ^6^, ^24^, ^25^ associated with higher recurrence and lower long‐term survival. Among horses, two of three survivors and two of four non‐survivors had EMS, whereas in ponies all non‐survivors and one of four survivors were endocrinopathic. Persistent hyperinsulinemia and impaired lamellar repair in horses with EMS or PPID likely contribute to poorer outcomes despite effective mechanical unloading.^26^, ^27^ These findings reinforce the importance of concurrent endocrine management alongside surgical intervention.

Although the overall surgical complication rate was low, all cases of wound dehiscence occurred in horses with PPID, consistent with previously described delayed healing and altered immune function associated with chronic hypercortisolemia in this population.^28^, ^29^

### 4.1 Study limitations

The retrospective design of the present study and absence of a control group preclude definitive comparison with other tenotomy techniques or postoperative protocols. The small sample size, variable disease duration and etiology, and owner‐driven case selection introduce potential bias and confounding factors. Inconsistent follow‐up intervals and reliance on referring veterinarians for later assessments may have added interobserver variability. Routine monitoring of renal parameters was not consistently performed; although not always feasible in clinical practice, serial renal evaluation would represent a higher standard of care when administering long‐term nonsteroidal anti‐inflammatory drugs (NSAIDs) and should be considered in future prospective studies. Despite these limitations, use of standardized surgical and farriery protocols across all cases strengthens internal consistency and supports the observed clinical trends.

### 4.2 Clinical significance

High‐metacarpal DDFT tenotomy performed proximal to the accessory ligament, combined with immediate orthotic supportusing Steward clogs, can provide effective P3 realignment while maintaining DIPJ stability. The approach expands available surgical options for managing refractory laminitis and supports a shift toward techniques that compromise between mechanical unloading and preservation of DIPJ integrity.

## AUTHOR CONTRIBUTIONS

Hargitaiova K, DVM: Conceptualization, data curation, formal analysis, project administration, resources, visualization, writing–original draft preparation, writing–review and editing. Maleas G: Conceptualization, investigation, methodology, supervision, writing–original draft preparation, writing–review and editing. Both authors have read and approved the final version of the manuscript and agree to be accountable for all aspects of the work.

## CONFLICT OF INTEREST STATEMENT

The authors declare no conflicts of interest related to this report.

## Data Availability

The data supporting the findings of this study are available from the corresponding author upon reasonable request.

## References

[1] Orsini JA , Parsons CS , Capewell L , Smith G . Prognostic indicators of poor outcome in horses with laminitis at a tertiary care hospital. Can Vet J. 2010;51(6):623‐628. 20808574 PMC2871359

[2] Patterson‐Kane JC , Karikoski NP , McGowan CM . Paradigm shifts in understanding equine laminitis. Vet J. 2018;231:33‐40. doi:10.1016/j.tvjl.2017.11.011 29429485

[3] Hood DM , Grosenbaugh DA , Slater MR . Vascular perfusion in horses with chronic laminitis. Equine Vet J. 1994;26(3):191‐196. doi:10.1111/j.2042-3306.1994.tb04368.x 8542837

[4] van Eps AW , Pollitt CC . Equine laminitis induced with oligofructose. Equine Vet J. 2010;38(3):203‐208. doi:10.2746/042516406776866327 16706272

[5] Wylie CE , Collins SN , Verheyen KLP , Newton JR . Risk factors for equine laminitis: A case‐control study conducted in veterinary‐registered horses and ponies in Great Britain between 2009 and 2011. Vet J. 2013;198(1):57‐69. doi:10.1016/j.tvjl.2013.08.028 24070987

[6] Horn R , Bamford NJ , Afonso T , et al. Factors associated with survival, laminitis and insulin dysregulation in horses diagnosed with equine pituitary pars intermedia dysfunction. Equine Vet J. 2019;51(4):440‐445. doi:10.1111/evj.13041 30417404

[7] van Eps AW , Pollitt CC . Equine laminitis model: lamellar histopathology seven days after induction with oligofructose. Equine Vet J. 2009;41(8):735‐740. doi:10.2746/042516409X444953 20095219

[8] Gutierrez‐Nibeyro SD , McCoy AM , Selberg KT . Recent advances in conservative and surgical treatment options of common equine foot problems. Vet J. 2018;237:9‐15. doi:10.1016/j.tvjl.2018.05.003 30089549

[9] Hunt RJ . A retrospective evaluation of laminitis in horses. Equine Vet J. 1993;25(1):61‐64. doi:10.1111/j.2042-3306.1993.tb02903.x 8422888

[10] Eastman TG , Honnas CM , Hague BA , Moyer W , von der Rosen HD . Deep digital flexor tenotomy as a treatment for chronic laminitis in horses: 35 cases (1988‐1997). J Am Vet Med Assoc. 1999;214(4):517‐519. 10029854

[11] Auer JA , Stick JA , Kummerle JM , Prange T . Equine Surgery. 5th ed. Elsevier; 2019. doi:10.1016/C2015-0-05672-6

[12] de Oliviera Dearo AC , Rosa VBB , Reichmann P , de Oliviera ML . Effects of two different deep digital flexor tenotomy techniques on distal articular angles of equine cadaver forelimbs. Cienc Rural. 2012;42(11):2005‐2010.

[13] Denoix JM . Functional anatomy of tendons and ligaments in the distal limbs (manus and pes). Vet Clin North Am Equine Pract. 1994;10(2):273‐321. 7987720 10.1016/s0749-0739(17)30358-9

[14] Cripps PJ , Eustace RA . Factors involved in the prognosis of equine laminitis in the UK. Equine Vet J. 1999;31(5):433‐442. doi:10.1111/j.2042-3306.1999.tb03845.x 10505961

[15] Steward ML . The use of the wooden shoe (Steward Clog) in Treating Laminitis. Vet Clin North Am Equine Pract. 2010;26(1):207‐214. doi:10.1016/j.cveq.2009.12.002 20381748

[16] D'Arpe L , Bernardini D . Digital Venography in Horses and Its Clinical Application in Europe. Vet Clin North Am Equine Pract. 2010;26(2):339‐359. doi:10.1016/j.cveq.2010.06.006 20699179

[17] Rucker A . Equine Venography and Its Clinical Application in North America. Vet Clin North Am Equine Pract. 2010;26(1):167‐177. doi:10.1016/j.cveq.2009.12.008 20381745

[18] Eustace RA , Caldwell MN . Treatment of solar prolapse using the heart bar shoe and dorsal hoof wall resection technique. Equine Vet J. 1989;21(5):370‐372. doi:10.1111/j.2042-3306.1989.tb02694.x 2776725

[19] Ritmeester AM , Blevins WE , Ferguson DW , Adams SB . Digital perfusion, evaluated scintigraphically, and hoof wall growth in horses with chronic laminitis treated with egg bar‐heart bar shoeing and coronary grooving. Equine Vet J Suppl. 1998;26:111‐118. doi:10.1111/j.2042-3306.1998.tb05129.x 9932101

[20] Morrison S . Long‐term Prognosis Using Deep Digital Flexor Tenotomy and Realignment Shoeing for Treatment of Chronic Laminitis. J Equine Vet Sci. 2011;31(2):89‐96. doi:10.1016/j.jevs.2010.12.008

[21] Zetterström SM , Boone LH , Farag R , Weimar WH , Caldwell FJ . Effect of single and double hemitenotomy on equine deep digital flexor tendon length and strength in experimental load challenges. Vet Surg. 2022;51(7):1153‐1160. doi:10.1111/vsu.13808 35437771

[22] De Gasperi D , El Azzi MS , Martins JPN , Brounts SH . Ex vivo study shows novel, rapid, suture‐free tenotomy technique for the equine deep digital flexor tendon. Am J Vet Res. 2024;85(2):1‐6. doi:10.2460/ajvr.23.09.0215 38109844

[23] O'Grady SE . Farriery for chronic laminitis. Vet Clin North Am Equine Pract. 2010;26(2):407‐423. doi:10.1016/j.cveq.2010.04.008 20699184

[24] Karikoski NP , Patterson‐Kane JC , Singer ER , McFarlane D , McGowan CM . Lamellar pathology in horses with pituitary pars intermedia dysfunction. Equine Vet J. 2016;48(4):472‐478. doi:10.1111/evj.12450 25869529

[25] Karikoski NP , Horn I , McGowan TW , McGowan CM . The prevalence of endocrinopathic laminitis among horses presented for laminitis at a first‐opinion/referral equine hospital. Domest Anim Endocrinol. 2011;41(3):111‐117. doi:10.1016/j.domaniend.2011.05.004 21696910

[26] Asplin KE , Sillence MN , Pollitt CC , McGowan CM . Induction of laminitis by prolonged hyperinsulinaemia in clinically normal ponies. Vet J. 2007;174(3):530‐535. doi:10.1016/j.tvjl.2007.07.003 17719811

[27] Frank N , Geor RJ , Bailey SR , Durham AE , Johnson PJ . Equine Metabolic Syndrome. J Vet Intern Med. 2010;24(3):467‐475. doi:10.1111/j.1939-1676.2010.0503.x 20384947

[28] Miller C , Utter ML , Beech J . Evaluation of the Effects of Age and Pituitary Pars Intermedia Dysfunction on Corneal Sensitivity in Horses. Am J Vet Res. 2013;74:1030‐1035. 23802675 10.2460/ajvr.74.7.1030

[29] Kirkwood NC , Hughes KJ , Stewart AJ . Pituitary Pars Intermedia Dysfunction (PPID) in Horses. Vet Sci. 2022;9(10):556. doi:10.3390/vetsci9100556 36288169 PMC9611634

